# Benefits and challenges of multi-delay arterial spin labeling in clinical practice: measuring perfusion and cerebrovascular reactivity in intracranial steno-occlusive disease

**DOI:** 10.1186/s13244-025-02077-4

**Published:** 2025-09-18

**Authors:** Simone M. Uniken Venema, Alex Bhogal, Jan Willem Dankbaar, H. Bart van der Worp, Jeroen Hendrikse, Bart van der Zwan, Kees Braun, Jeroen C. W. Siero, Pieter T. Deckers

**Affiliations:** 1 Department of Radiology and Nuclear Medicine, University Medical Center Utrecht, Utrecht, The Netherlands; 2 Translational Neuroimaging Group, Department of Radiology and Nuclear Medicine, Center for Image Sciences, University Medical Center Utrecht, Utrecht, The Netherlands; 3 Department of Neurology and Neurosurgery, University Medical Center Utrecht, Utrecht, The Netherlands; 4 Gradient Group, Department of Radiology and Nuclear Medicine, Center for Image Sciences, University Medical Center Utrecht, Utrecht, The Netherlands

**Keywords:** Arterial spin labeling, Cerebrovascular reactivity, Arterial transit time, Moyamoya, Magnetic resonance

## Abstract

**Abstract:**

Magnetic resonance imaging (MRI) techniques have now widely replaced positron emission tomography (PET) as the modality of choice to assess cerebrovascular reactivity (CVR) and other hemodynamic parameters in intracranial steno-occlusive disease (ISOD), such as moyamoya vasculopathy (MMV). Therefore, radiologists should be aware of the choices in imaging techniques and potential pitfalls in the imaging interpretation. We developed a protocol based on multi-delay arterial spin labeling (ASL), with which, since its implementation in routine clinical practice in 2018, approximately 100 patients have been evaluated for CVR and other parameters. The protocol demonstrates clinical feasibility and utility, allowing detailed cerebral hemodynamic evaluations of individual patients that are useful for clinical decision-making. While multi-delay ASL offers benefits compared to single-delay ASL in patients with arterial transit delays, it is not completely insensitive to transit times, and further methodological improvements could mitigate this issue in the near future. Potential pitfalls in imaging acquisition and interpretation include artefacts due to motion, susceptibility, and misalignment in imaging registration, inadequate labeling, and the effects of anesthesia on CVR. This work serves as a practical guide for clinicians, radiologists, and MRI experts seeking to implement these advanced imaging methods in their institutions.

**Critical relevance statement:**

Our MRI protocol, based on multi-delay ASL with a vascular challenge of acetazolamide, can be successfully used for hemodynamic assessments of patients with ISOD in clinical settings.

**Key Points:**

- We developed and implemented a protocol using acetazolamide-augmented multi-delay ASL for hemodynamic assessments in patients with steno-occlusive disease.
- Obtaining hemodynamic maps from ASL source data requires pre- and post-processing steps using customized toolboxes.
- CVR, if decreased, indicates hemodynamic compromise.
- Multi-delay ASL is not completely insensitive to delayed arterial transit, and technical improvements are needed to mitigate this.

**Graphical Abstract:**

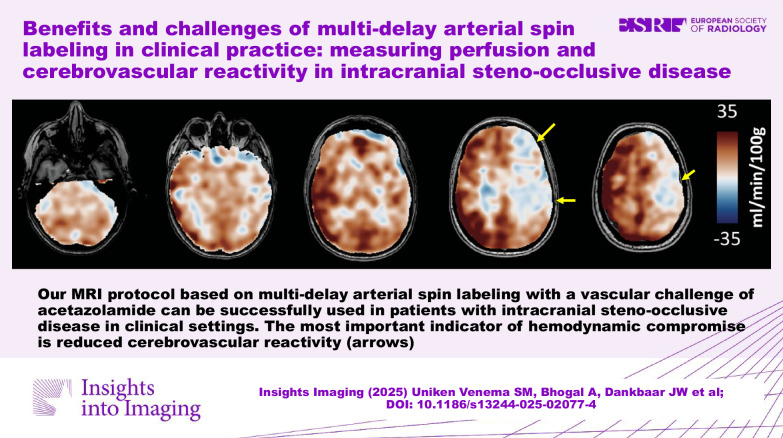

## Introduction

Patients with intracranial steno-occlusive disease (ISOD) comprise a heterogeneous group with varying underlying etiologies, such as atherosclerotic stenosis and moyamoya vasculopathy (MMV). In patients with ISOD, post-stenotic vasodilation occurs in an attempt to compensate for the reduction in cerebral blood flow (CBF). This response may be attenuated in tissue that has reached the limits of the vasodilatory capacity, a phenomenon that can be quantified by measuring the cerebrovascular reactivity (CVR): the CBF change in response to a vasodilatory stimulus. Cerebrovascular ‘steal’ is a paradoxical phenomenon whereby CBF reduces after a vasodilatory stimulus, leading to a negative CVR [1, 2]. Reduced or negative CVR is directly related to stroke risk in patients with ISOD [3, 4].

Clinical symptoms, hemodynamic data, and angiographic imaging guide decisions about neurosurgical intervention. Until recently, CVR was commonly assessed with a [^15^O]-H_2_O positron emission tomography (PET) scan with an acetazolamide challenge [5, 6]. This method has several disadvantages: radiation exposure, high costs, and limited accessibility due to the requirement for an on-site cyclotron. Therefore, certain magnetic resonance imaging (MRI) techniques, especially BOLD and arterial spin labeling (ASL) MRI, have emerged as promising non-invasive tools to assess CVR [7]. ASL uses magnetically labeled arterial blood water as an endogenous tracer to measure CBF. The magnetization of inflowing arterial blood is inverted by applying a radiofrequency pulse to a plane at the level of cervical arteries, typically 8–10 cm below the circle of Willis, encompassing the extracranial segments of the internal and common carotid arteries, as well as the vertebral arteries [8]. Blood is magnetically labeled during a predefined labeling duration (typically 1.5–2.0 s), after which images are acquired following a post-labeling delay (PLD). CBF is calculated by subtracting labeled and control images and quantified (in mL/100 g/min) using a calibration image (M_0_). When combined with a vasodilatory stimulus, ASL-CVR enables direct quantification of CVR, defined as the change in absolute CBF, by subtracting the pre-stimulus CBF map from the post-stimulus map. This technique has been compared to [^15^O]-H_2_O PET-CT, albeit in small studies, and showed good correlation [9–11].

A major challenge in conventional ASL using a fixed label duration and a single PLD is its sensitivity to the arterial transit time (ATT). This is especially relevant in patients with ISOD, who often have abnormally long ATT in regions sustained by collateral flow. If the PLD is shorter than the ATT, magnetically labeled blood will remain within the arteries, creating linear bright spots on imaging called arterial transit artifact (ATA) [12, 13]. Regions supplied by these arteries will show an underestimation of the CBF [9]. Moreover, vasoactive stimuli themselves can modulate ATT and potentially compromise CBF and CVR quantification, with single-PLD ASL [14]. Alternatively, the PLD and labeling duration can be varied over repeated acquisitions, known as multi-delay ASL and time-encoded ASL [15]. This method more accurately estimates CBF and has the additional advantage of quantifying other hemodynamic parameters, such as ATT, which has been shown to be a useful marker of pathology in itself [16, 17]. Continued technical developments in multi-delay ASL have substantially increased its applicability in clinical practice, as well as research settings, leading the ISMRM Perfusion Study Group to recommend it for hemodynamic evaluation in patients with ISOD [18].

In this paper, we describe our clinical workflow and protocol for the hemodynamic assessment of patients with ISOD, and we critically discuss the advantages and challenges of our approach. Our goal is to provide guidance for centers and individual radiologists wishing to expand their expertise with advanced hemodynamic imaging.

## Materials and methods

### Study design, study population, and ethics

This research is a sub-study of a larger retrospective cohort study, which aimed to evaluate the clinical use of advanced hemodynamic imaging in patients with ISOD. Since 2018, patients were consecutively evaluated using the protocol described below, and data were collected retrospectively. While the present study does not pose a novel research question, it seeks to share our experience with the technical aspects and clinical implementation of the protocol in order to inform similar efforts at other centers. The Institutional Review Board of the University Medical Center Utrecht declared that the Medical Research Involving Human Subjects Act does not apply to the present research, since all study measures were part of routine clinical practice. Inclusion criteria were: (1) imaging diagnosis of ISOD, irrespective of the underlying cause, (2) clinical indication to undergo hemodynamic imaging at the University Medical Center Utrecht, and (3) written informed consent by the patient or their legal representative (i.e., parent or guardian) for the use of data. Exclusion criteria were: (1) inability to undergo MRI (e.g., due to claustrophobia) and (2) refusal to provide written informed consent. There was no age restriction: both adult and pediatric patients could be included.

### Preparation

The patient is admitted on the day of the scan and instructed not to drink caffeine-containing beverages beforehand [19].

Acetazolamide is prepared on the ward (dose of 20 mg/kg, with a maximum of 1 g, dissolved in 30 cc 0.9% NaCl solution); the nurse is present during the (automated) injection of the acetazolamide during the MRI in case of adverse events. Afterwards, the patient is monitored on the ward for several hours.

For children, anesthesia during scanning may reduce the risk of scan failure due to motion artifacts or an incomplete protocol. Anesthesia is induced with propofol or sevoflurane at the discretion of the pediatric anesthesiologist, and breathing during anesthesia is managed using a laryngeal mask. More details on the anesthesia protocol, and its influence on measured CVR, are described separately [20].

### Image acquisition

Participants are scanned on a 3-Tesla MRI scanner (Achieva, Philips Medical Systems, Best, The Netherlands) using a 32-channel receive coil. Image acquisition is done entirely by radiographers.

The ASL labeling is planned using a rapid phase contrast angiography scan, with the plane perpendicular to major arteries (Fig. S1). CBF measurements are obtained before and after acetazolamide using multi-delay ASL with the following parameters: look-locker readout, pseudo-continuous ASL, multi-slice EPI, 5 PLDs (1206–3480 ms), 16 slices, 24 volumes, 3.75 mm voxelsize and 7 mm slice thickness, TR/TE = 6 s/11 ms, flip angle = 25° (see Fig. S2 for an example of raw ASL data). An intravenous injection of acetazolamide (flow rate of 0.3 cc/s) is started upon completion of the first multi-delay ASL. Once the (presumed) maximum effect of acetazolamide is reached (15.5 min), a second MD-ASL scan with identical parameters as the baseline scan is acquired. The timing of the presumed maximum effect was based on a previous study, which assessed the time course of acetazolamide-induced vasodilation in patients with internal carotid artery stenosis [21].

Other sequences in the protocol are described in Table S3.

### Image pre-processing and analysis

Pre-processing steps are performed using the Oxford Centre for Functional MRI of the Brain Software Library (version 6.0). Tissue segmentation into gray matter, white matter, and cerebrospinal fluid are performed on the T1 imaging using the FSL Automated Segmentation Tool.

Multi-delay ASL data are processed and analyzed with an in-house developed pipeline script in MATLAB, the ClinicalASL toolbox (available at: https://github.com/JSIERO/ClinicalASL). A T1-weighted image is generated based on the M0 images from each PLD, which is used for subsequent image registration. FSL BASIL is used to generate quantitative CBF and ATT maps, both pre- and post-acetazolamide. CVR maps are computed by subtracting post-acetazolamide CBF maps from the pre-acetazolamide CBF maps. As such, ASL CVR is defined as the CBF change from baseline to post-acetazolamide. CBF maps generated from the 2nd through 5th PLD are used for the calculation of CVR. Additional pre- and post-acetazolamide CBF maps are generated using the 1st and 2nd PLD only, from which ATAs can be assessed. The resulting hemodynamic maps generated from ASL data are compiled in a single PDF file showing sixteen axial slices of the brain for each map (Fig. S4).

## Results

### Patients

**Fig. 1:**
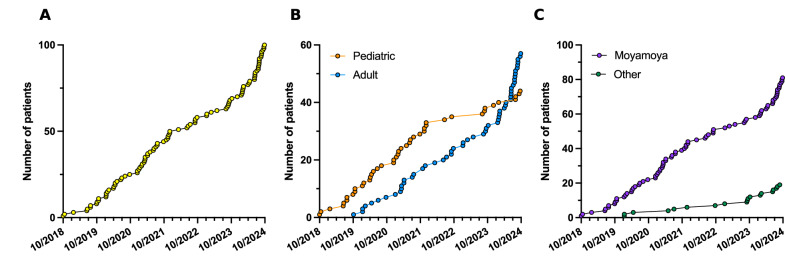
Total number of hemodynamic MRIs requested for clinical purposes since the implementation of the clinical protocol in 2018 ((**A**) all patients; (**B**) pediatric patients vs adult patients, and (**C**) patients with moyamoya vs other etiology)

In the University Medical Center Utrecht, multi-delay ASL perfusion imaging, combined with a vasodilatory challenge with acetazolamide, has been integrated into the routine hemodynamic evaluation of patients with ISOD since 2018 and so far around 100 patients have been scanned (Fig. 1A). The total number of scans requested by clinicians has seen a gradual rise over time, which is mostly attributed to the rise in scans requested for adult patients (Fig. 1B) and for diagnoses other than MMV (most frequently atherosclerotic carotid stenosis, Fig. 1C). The most common indications for undergoing the MRI scan were a new transient ischemic attack or infarction in a patient with known ISOD (48%), pre-operative evaluation for bypass surgery (27%), unclear symptoms (12%) and postoperative evaluation (11%). In two patients, the indication for hemodynamic evaluation was the incidental finding of ISOD on imaging performed for another indication.

Additional patient characteristics are provided in Table S5.

### Adverse events

A total of four adverse events (~4% of total examinations) following the acetazolamide challenge were reported. One child had a headache shortly after the acetazolamide injection. One adult and one child suffered from paresthesias, likely as a result of hyperventilation induced by acetazolamide. One child was admitted during one night because of mild metabolic acidosis caused by acetazolamide (without clinical symptoms). All of these events recovered spontaneously. There were no reports of ischemic symptoms following acetazolamide.

### Artifacts

Due to the dynamic nature of acquisition, hemodynamic imaging is inherently susceptible to artifacts, potentially causing misinterpretation. In our patient population, 40.6% had no artifacts in the hemodynamic ASL-based maps, 34.8% had minor artifacts that did not substantially interfere with image interpretation (artifacts from surgical clips, a small registration artifact or top slices missing) and 11.6% had suboptimal imaging, usually due to motion artifacts, anesthesia or extensive artifacts from clips (artifacts somewhat interfered with image interpretation, but these results were still useful in clinical practice). Finally, 13.0% had poor results that were not useful in clinical practice, frequently caused by large motion artifacts that could not be corrected with postprocessing, multiple simultaneous artifacts, such as motion and extensive artifacts from clips, or failed labeling.

### Interpretation and reporting

ASL-derived hemodynamic maps (pre- and post-acetazolamide CBF, ATT, and ATA, and calculated CVR maps) and structural imaging are available in the picture archiving and communication system (PACS) (Fig. S4).

The interpretation includes assessing the success of the vasodilatory challenge. CVR should increase in the cerebellum, since the posterior circulation is usually unaffected in ISOD. If this is not the case, the underlying cause should be determined, and whether this affects the assessment of hemispheric CVR. The most common causes are: tissue defect in the cerebellum; significant stenosis in vertebral arteries or basilar artery; the use of anesthesia; incorrect or insufficient administration of acetazolamide (for instance, due to extravasation).

**Fig. 2:**
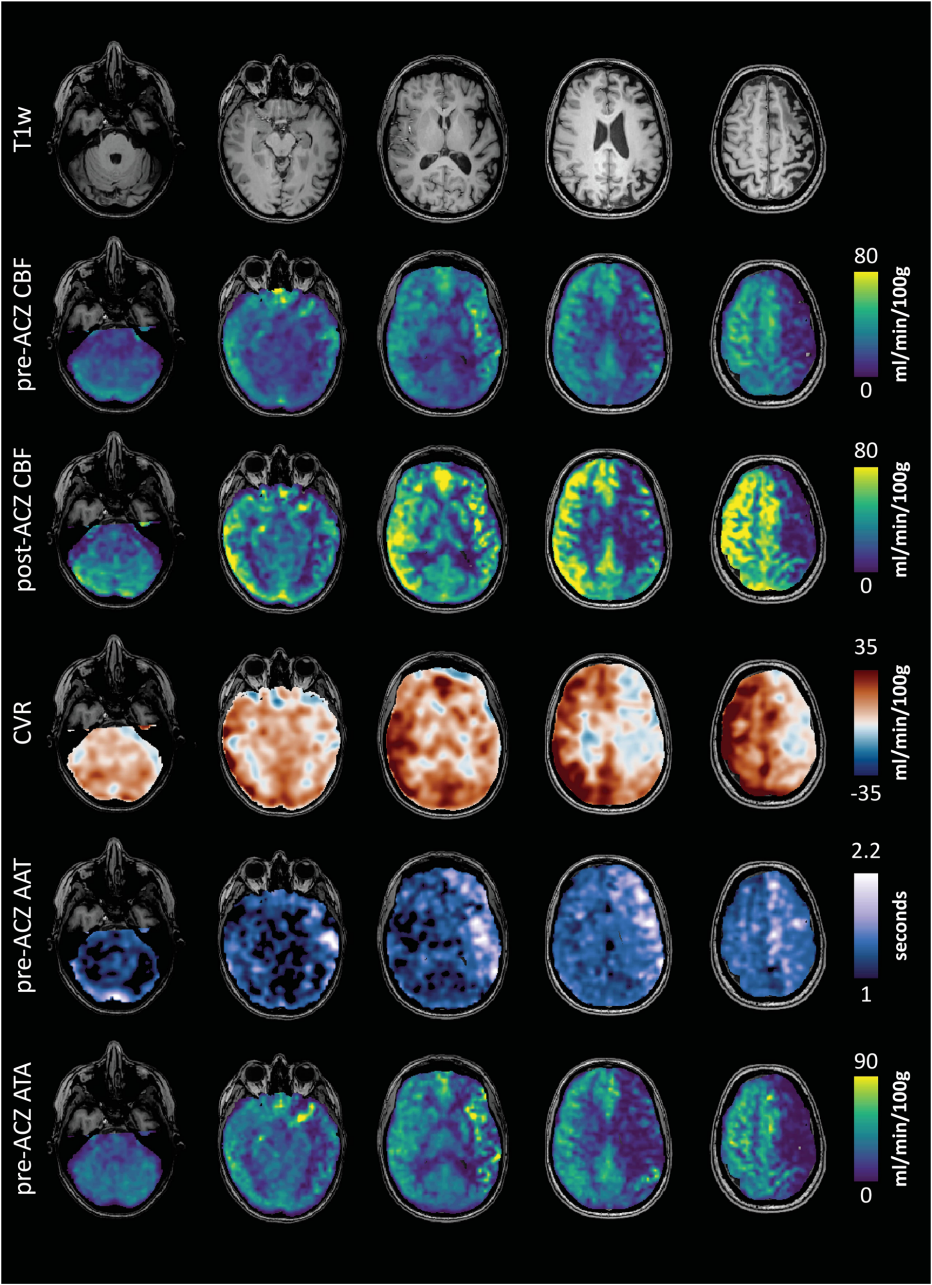
Hemodynamic maps derived from ASL, including pre-acetazolamide CBF, post-acetazolamide CBF, CVR, pre-acetazolamide arterial arrival time (AAT), and pre-acetazolamide arterial transit artifacts (ATAs). In clinical practice, each map consists of sixteen (caudal to cranial) axial ASL-MRI slices (see Fig. S3). The pre-acetazolamide CBF maps show a lower CBF in the left MCA territory, with prolonged AAT, as well as multiple ATAs in the corresponding territory. The post-CBF map shows a normal increase in CBF in the cerebellum and right hemisphere, but no response in the left hemisphere. Hence, the CVR map (calculated by subtracting the pre-acetazolamide CBF map from the post-acetazolamide CBF map) indicates a large are of steal in the left hemisphere. This is caused by a severe stenosis of the left M1 on MRA (not shown)

When interpreting the hemodynamic maps, rather than focusing on absolute values, asymmetries should be compared between the left and right hemispheres and between vascular territories. Hemodynamically compromised hemispheres typically exhibit reduced baseline CBF and delayed ATT, manifested as ATAs. Following acetazolamide, the affected region may show no increase or even a decrease in CBF, indicative of cerebral steal. An example is provided in Fig. 2: the pre-acetazolamide CBF maps show a lower CBF in the left middle cerebral artery (MCA) territory, with prolonged AAT, as well as multiple ATAs in the corresponding territory. The post-CBF map shows a normal increase in CBF in the cerebellum and right hemisphere, but no response in the left hemisphere. Hence, the CVR map (calculated by subtracting the pre-acetazolamide CBF map from the post-acetazolamide CBF map) indicates a large are of steal in the left hemisphere. This is caused by a severe stenosis of the left M1 on MRA (not shown).

Generalized atrophy or focal tissue loss can appear as low or negative CVR due to minimal CBF in affected areas. CVR should thus be interpreted in the context of structural images and baseline CBF.

Suggested steps to obtain a structured report are depicted in Table 1.

**Table 1:** Suggested steps to obtain a structured report

Step 1: Assessment of angiography and available structural imaging.
- Determine and report location(s) of old infarcts, DWI lesions and (micro-) hemorrhage.
- Determine and report location(s), severity, and progression of intracranial stenoses.
- Determine whether an STA-MCA bypass is present (should also be provided in the clinical information) and assess the patency of the bypass.
- Assess the extent of collateral circulation and any change therein in case of a (recent) bypass surgery.
Step 2: Assessment of the success of the vasoactive challenge by assessing CVR in the cerebellum.
Step 3: Assessment of regional differences in baseline CBF, ATT, and ATAs.
Step 4: Identify areas with regional differences in CVR and areas with cerebral steal (negative CVR).
Step 5: Reporting of artifacts, if present, that limit the reliability of the interpretation.
Step 6: The conclusion should contain:
- Any new, relevant findings on structural imaging.
- Presence of regional differences in baseline CBF, ATT, and CVR, especially steal areas.

## Discussion

We present our experience with the acquisition, analysis, and interpretation of advanced hemodynamic MRI in the clinic. The cornerstone of our protocol is multi-delay ASL with a vasodilatory challenge of acetazolamide, as an alternative to [^15^O]-H_2_O PET-CT. Though multi-PLD ASL is not (yet) widely used in routine clinical practice, the technique gains increasing interest as it can account for the effect of variable ATT on CBF values and yields spatial information on ATT itself, providing additional hemodynamic information [17, 18]. Similarly, CVR is increasingly recognized as a marker of vascular dysfunction and has therefore been researched in the context of diseases other than ISOD, such as cerebral small vessel disease [22, 23], Alzheimer’s disease [24], subarachnoid hemorrhage [25], and acute ischemic stroke [26]. As such, CVR measurements and other hemodynamic parameters are likely to gain a place in routine MRI protocols for prognostic purposes or making treatment decisions. This underlines the need for (neuro)radiologists to become familiar with these techniques and the challenges accompanying them.

**Fig. 3:**
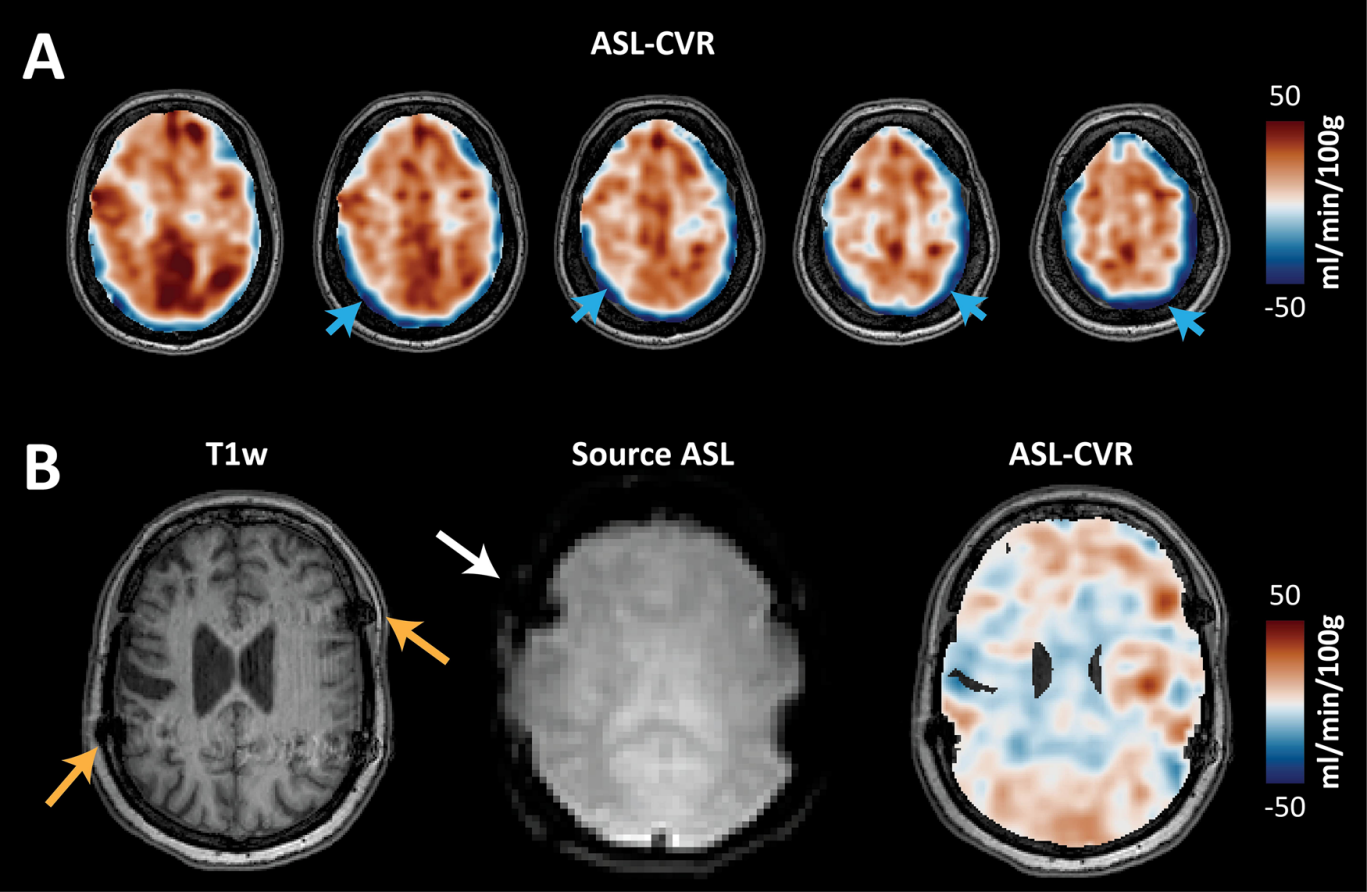
Common artifacts. **A** ASL-CVR map shows a rim of negative CVR (in blue) around the periphery of the brain. This is due to slight misregistration after aligning the post-acetazolamide image to the pre-acetazolamide image, rather than depicting a true steal phenomenon. **B** A patient who underwent neurosurgical intervention for both hemispheres has bilateral neurocranium clips, which distort the signal. The patient moved during the ASL. The ASL-CVR map is heterogeneous and shows scattered areas of lower CVR and steal, likely (partially) caused by motion artifacts

The ASL signal is inherently weak compared to the background tissue signal and requires multiple images to be averaged for acceptable CBF and ATT assessment. Since the labeling images are subtracted from the control images in ASL, the sequence is very sensitive to movement during that period, which can lead to inaccurate results (Fig. 3). To mitigate motion artifacts, background suppression is generally used, and motion correction can be applied as a postprocessing step (for instance, by removing label-control pairs showing abnormally high or low CBF), but these techniques are not suitable for extreme motion artifacts. To reduce the risk of motion in the acquisition phase (especially in pediatric patients), anesthesia is also an option. However, though the effect of acetazolamide combined with anesthetics is poorly investigated, it has been suggested that CVR under anesthesia is significantly underestimated [20]. This makes the interpretation of CVR challenging and possibly less reliable.

Multi-delay ASL presents clear advantages over single-delay ASL because it can partially compensate for the effects of prolonged bolus arrival time at the tissue and thus better estimate CBF in these cases. The ISMRM Perfusion Study Group therefore recommends the use of multi-delay ASL in patients with ISOD in a recent guidelines paper [18]. Nonetheless, a problem inherent to ASL is the fact that the signal of the labeled blood decays rapidly due to its relatively short T1. A long ATT therefore results in a low signal and poor CBF estimates. The other way around is also problematic: estimating ATT can be imprecise and biased in cases of low baseline CBF, as the signal, which under normal circumstances is already low in ASL, is even lower. Low CBF is often seen distal to an intracranial stenosis or occlusion, but it is also affected by other factors, such as age. A promising novel technique, known as variable-TR ASL, minimizes TR for each LD/PLD combination and shows a higher correlation to SPECT-based CBF than single delay pseudo-continuous ASL, notably for slow ATT regions [27]. This offers a more efficient method of acquisition, with the potential to fit more PLDs in the same scan time, thereby improving the robustness to subject motion and the signal-to-noise ratio to obtain better estimates of CBF and ATT. Other time-efficient techniques, such as time-encoded ASL, also known as Hademard-encoded ASL, or combinations of the former and latter in a hybrid fashion, are promising CBF and ATT mapping techniques for clinical applications [15, 18]. A step further, velocity-selective ASL offers a unique approach by labeling blood based on its velocity and is hence completely insensitive to transit delay effects [28]. Both variable-TR ASL and velocity-selective ASL are not yet readily available in clinical practice.

A few other potential issues are worth mentioning. First, the computed CBF image accuracy is affected by the labeling quality, which is manually planned (example provided in Fig. S1). If the labeling slab is incorrectly angled, the labeling will be less efficient, resulting in a lower ASL signal in the region supplied by that particular artery. This can lead to underestimation of CBF and should be suspected if CBF is abnormally low. Artefactual variation in the estimated CBF image can also be caused by strong susceptibility variations at the labeling plane location, e.g., caused by dental implants [13]. Second, ASL-CVR is calculated by subtracting the post-acetazolamide CBF map from the pre-acetazolamide CBF map after alignment by image registration. Because acetazolamide takes 12–15 min to take effect, some movement between these two scans is inevitable. If these images are not adequately aligned before the subtraction, this can lead to registration artifacts, often showing a ring of apparent hypo- or hyperperfused tissue around the upper part of the brain (Fig. 3).

Advantages of our multi-modal clinical protocol include increased accessibility, lower costs, fewer risks (with respect to ionizing radiation), and lower patient burden compared to PET. The use of acetazolamide offers convenience in the sense that radiographers can independently administer it without a specialized respiratory apparatus or MRI software modifications, making it suitable for routine clinical use. Moreover, acetazolamide has an excellent safety profile. Acetazolamide is a carbonic anhydrase inhibitor that penetrates the blood-brain barrier and causes metabolic acidosis, which in turn prompts vasodilation and induces a CBF increase. Theoretically, inducing vasodilation in a patient with impaired cerebral perfusion could induce symptoms of ischemia, based on the paradoxical steal effect. Though one case report described reversible pontine ischemia after an acetazolamide challenge [29], no ischemic strokes were reported in a study of > 1000 subjects [30]. The most commonly reported side effects of acetazolamide are transient, including paresthesias, malaise or fatigue, altered taste, polyuria, and headache [31, 32]. In our cohort, only four side effects were reported that all resolved spontaneously.

A limitation is the use of in-house developed scripts and offline postprocessing, which limits the wider application of this protocol in other practices. To our knowledge, there is no commercially available software that offers full on-scanner analysis and generation of the required hemodynamic maps. However, we are currently setting up an improved workflow that substantially reduces the amount of offline processing so that most analyses can be done on the scanner itself, and maps of CBF, CVR, and ATT can be sent directly to the PACS. Other future steps to further optimize hemodynamic assessment include OEF mapping using MRI [33] and further clinical implementation of improved ASL techniques (as described above), as well as 4D ASL-based MRA.

In summary, we have presented our clinical experience with an advanced MRI protocol using multi-delay ASL in combination with acetazolamide for the hemodynamic evaluation of patients with ISOD. Our protocol demonstrates clinical feasibility and utility, although radiologists and clinicians should be aware of potential pitfalls. A correct interpretation of hemodynamic images requires knowledge of clinical history and symptoms, imaging techniques, and structural imaging. Therefore, a multidisciplinary team involving MRI experts, radiologists, neurologists, and neurosurgeons is needed to make the right clinical decision.

## Supplementary information

ELECTRONIC SUPPLEMENTARY MATERIAL

## Data Availability

The customized MATLAB-based ClinicalASL toolbox for processing of ASL data is available at: https://github.com/JSIERO/ClinicalASL.

## Abbreviations

ASL: Arterial spin labeling
ATA: Arterial transit artefact
ATT: Arterial transit time
CBF: Cerebral blood flow
CVR: Cerebrovascular reactivity
ISOD: Intracranial steno-occlusive disease
MMV: Moyamoya vasculopathy
MRI: Magnetic resonance imaging
PET: Positron emission tomography
PLD: Post-labeling delay
