# Supplementary material for: Benefits and challenges of multi-delay arterial spin labeling in clinical practice: measuring perfusion and cerebrovascular reactivity in intracranial steno-occlusive disease
Source: Insights Imaging. 2025 Sep 18;16:197. doi: 10.1186/s13244-025-02077-4 (PMC12446178; doi:10.1186/s13244-025-02077-4)
Supplement: Supplementary file 1 — ELECTRONIC SUPPLEMENTARY MATERIAL [file 13244_2025_2077_MOESM1_ESM.pdf]

Benefits and Challenges of Multi-Delay Arterial Spin Labeling in Clinical Practice: Measuring Perfusion and Cerebrovascular Reactivity in Intracranial Steno-Occlusive Disease.

ELECTRONIC SUPPLEMENTARY MATERIAL

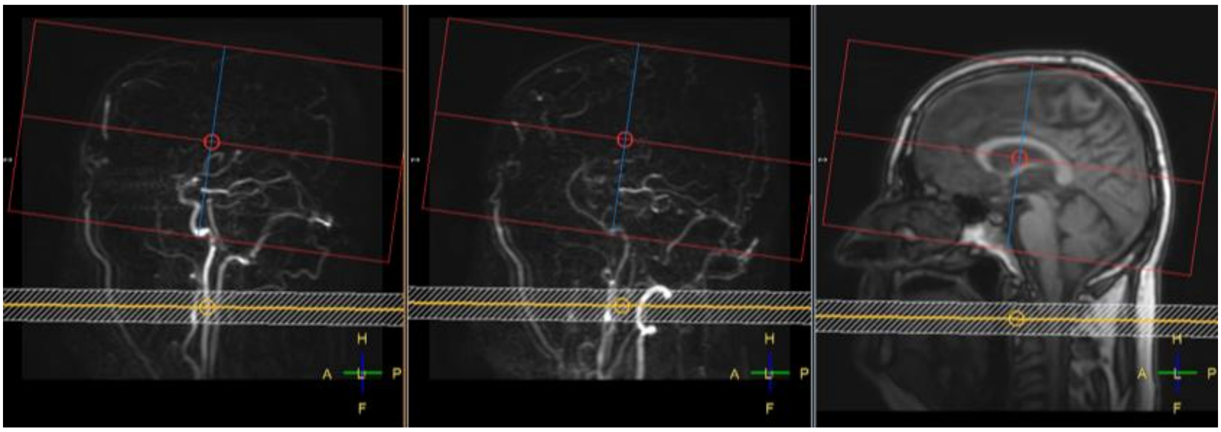

Figure S1: Correct placement of the labeling plane perpendicular to the cervical carotid vertebral arteries. Reduced labeling efficiency due to tortuous blood flow can be avoided using an angiographic survey to place the labeling plane (left part of the figure).

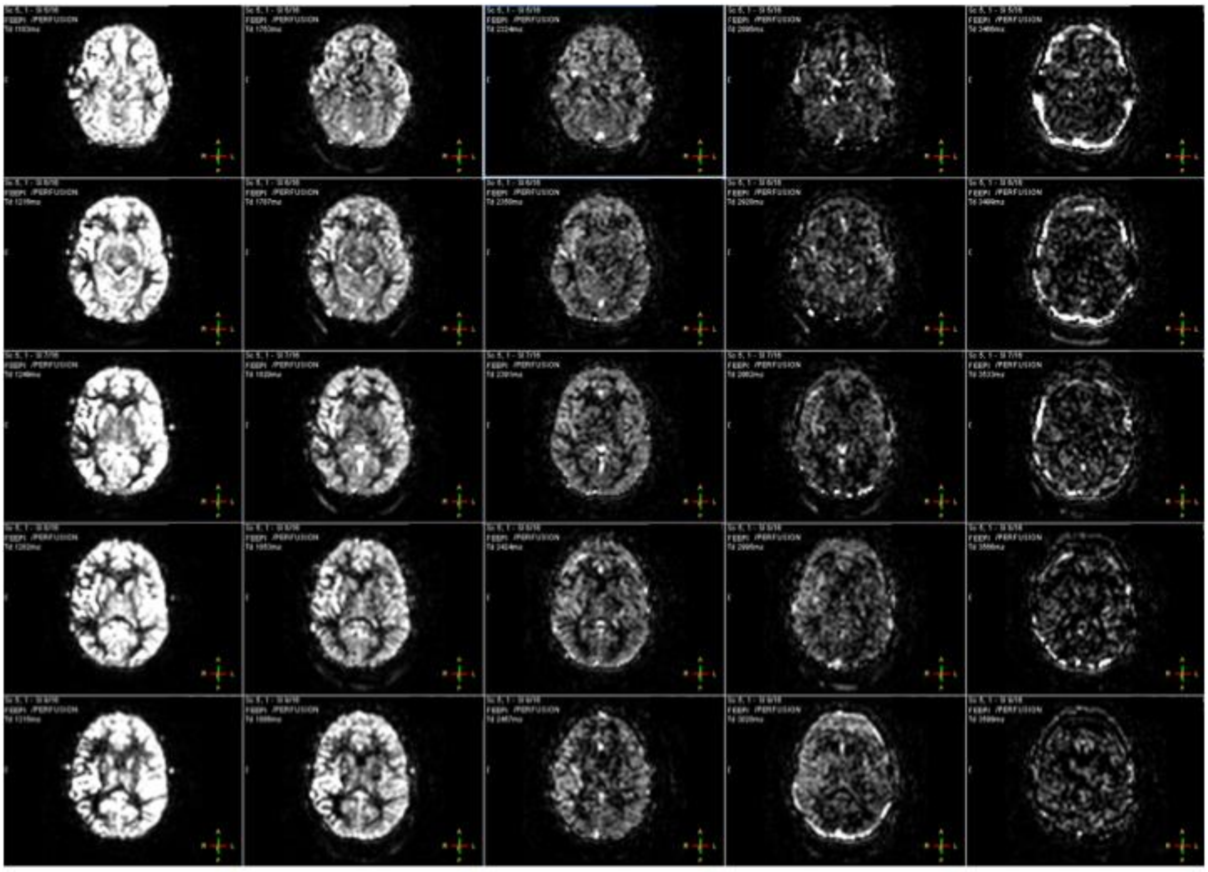

Figure S2: Multi-delay ASL source data with five post-labelling delays (left to right) showing five axial brain slices scanned in a caudal-to-cranial direction.

Table S3: Order and characteristics of sequences in the protocol.

Sequences	Plane/angulation	Duration
Survey PCA	Sagittal	Seconds
3D FLAIR	Transversal, angulated to anterior commissure-posterior commissure line	3 minutes
DW SPIR	Transversal, angulated to anterior commissure-posterior commissure line	4 minutes
MD ASL (pre-acetazolamide)	Transversal, angulated to anterior commissure-posterior commissure line	5 minutes
MRA 3D TOF	Angulated to the circle of Willis	5 minutes
SWI	Transversal, not angulated	5 minutes
T1 TFE 3D	Sagittal, angulated to anatomy	3 minutes
MD ASL (post-acetazolamide)	Transversal, angulated to anterior commissure-posterior commissure line	5 minutes

AC-PC = anterior commissure-posterior commissure; PCA = phase contrast angiography; FLAIR = fluid-attenuated inversion recovery; DW SPIR = diffusion weighted spectral presaturation with inversion recovery; MD ASL = multi-delay arterial spin labeling; MRA TOF = magnetic resonance angiography time-of-flight; SWI = susceptibility weighted imaging; TFE = turbo field echo.

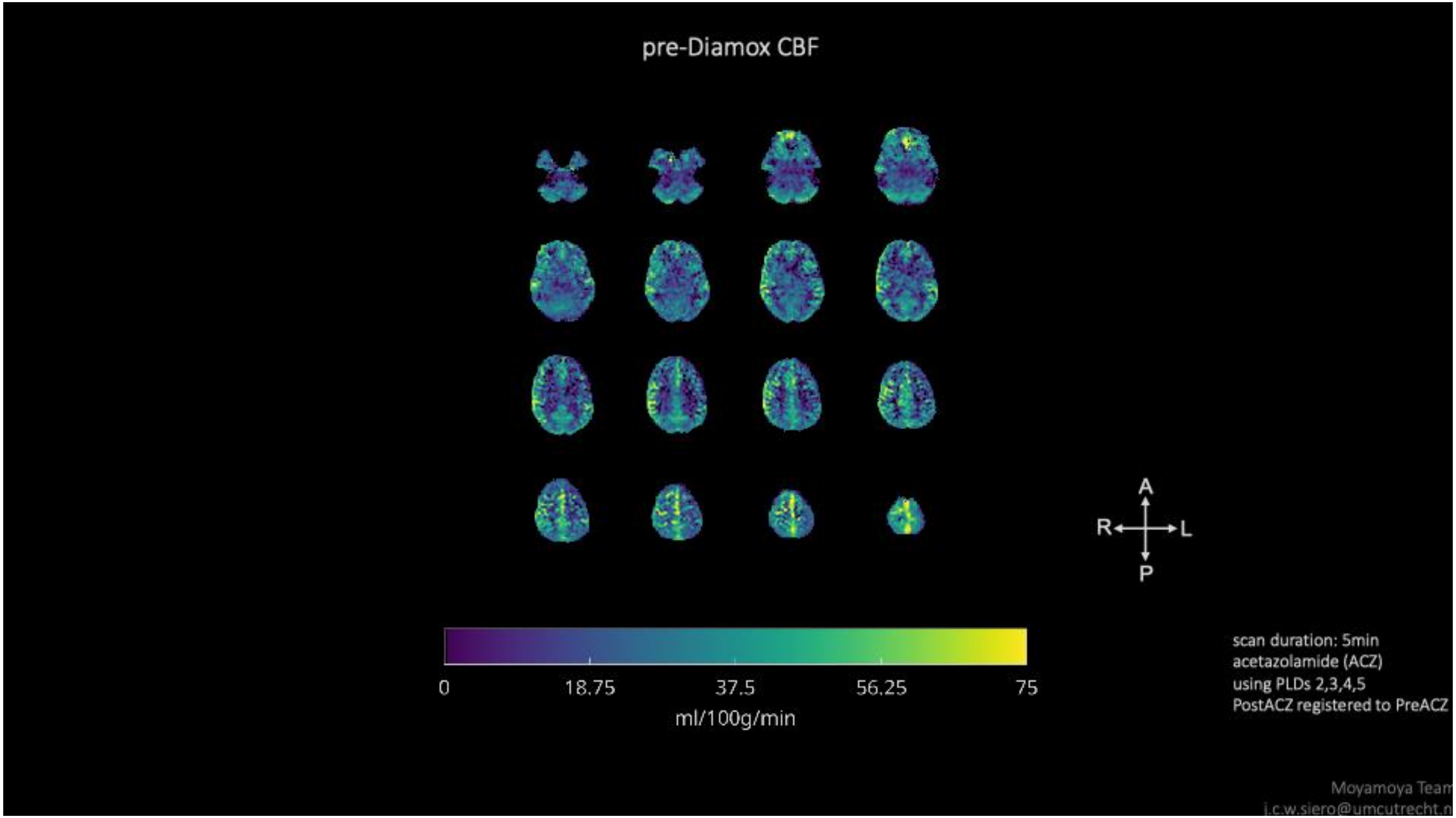

Figure S4: Example report containing multi-delay ASL results as received and to be interpreted by the radiologist and clinicians.

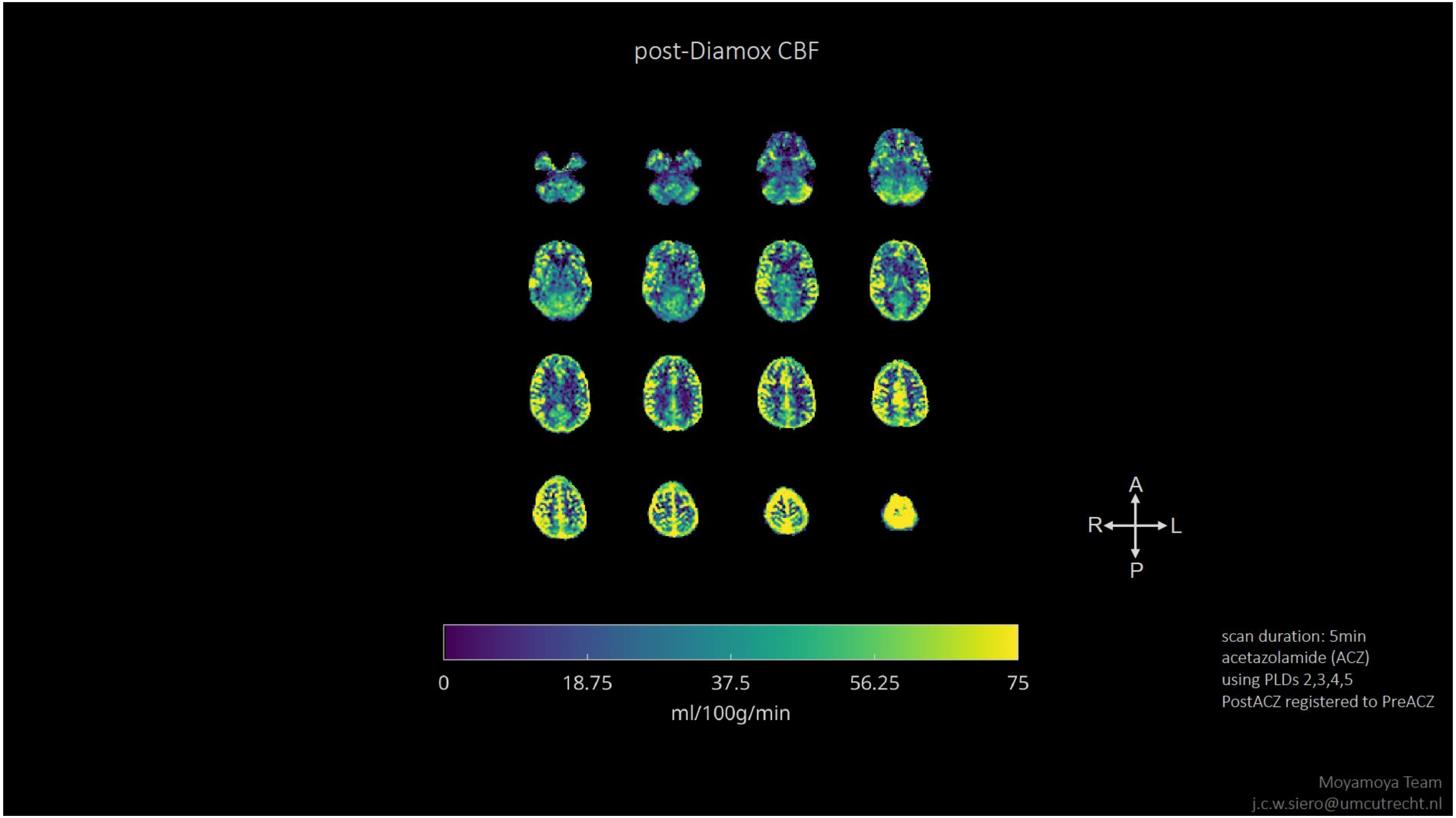

Figure S4 (continued).

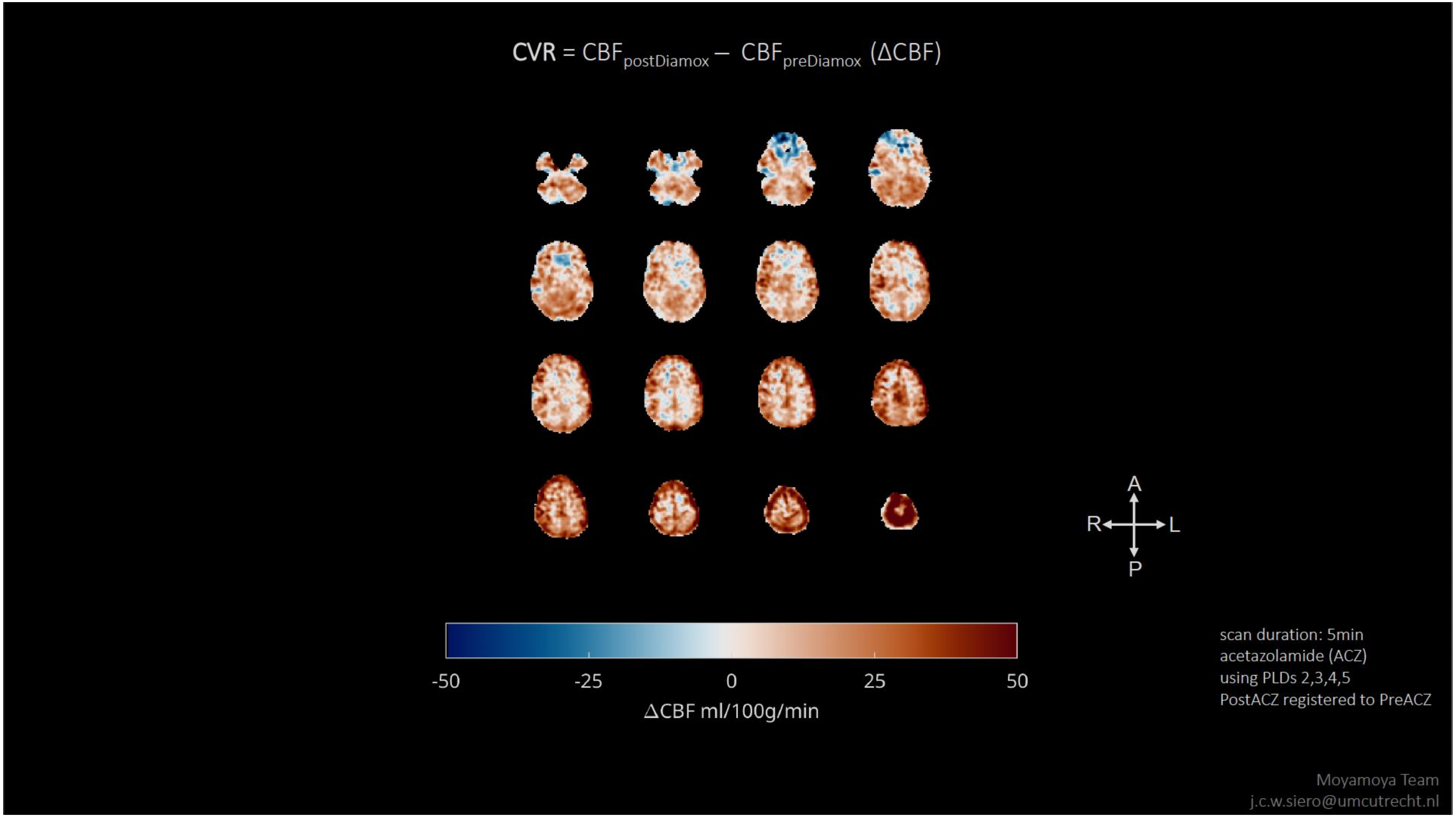

Figure S4 (continued).

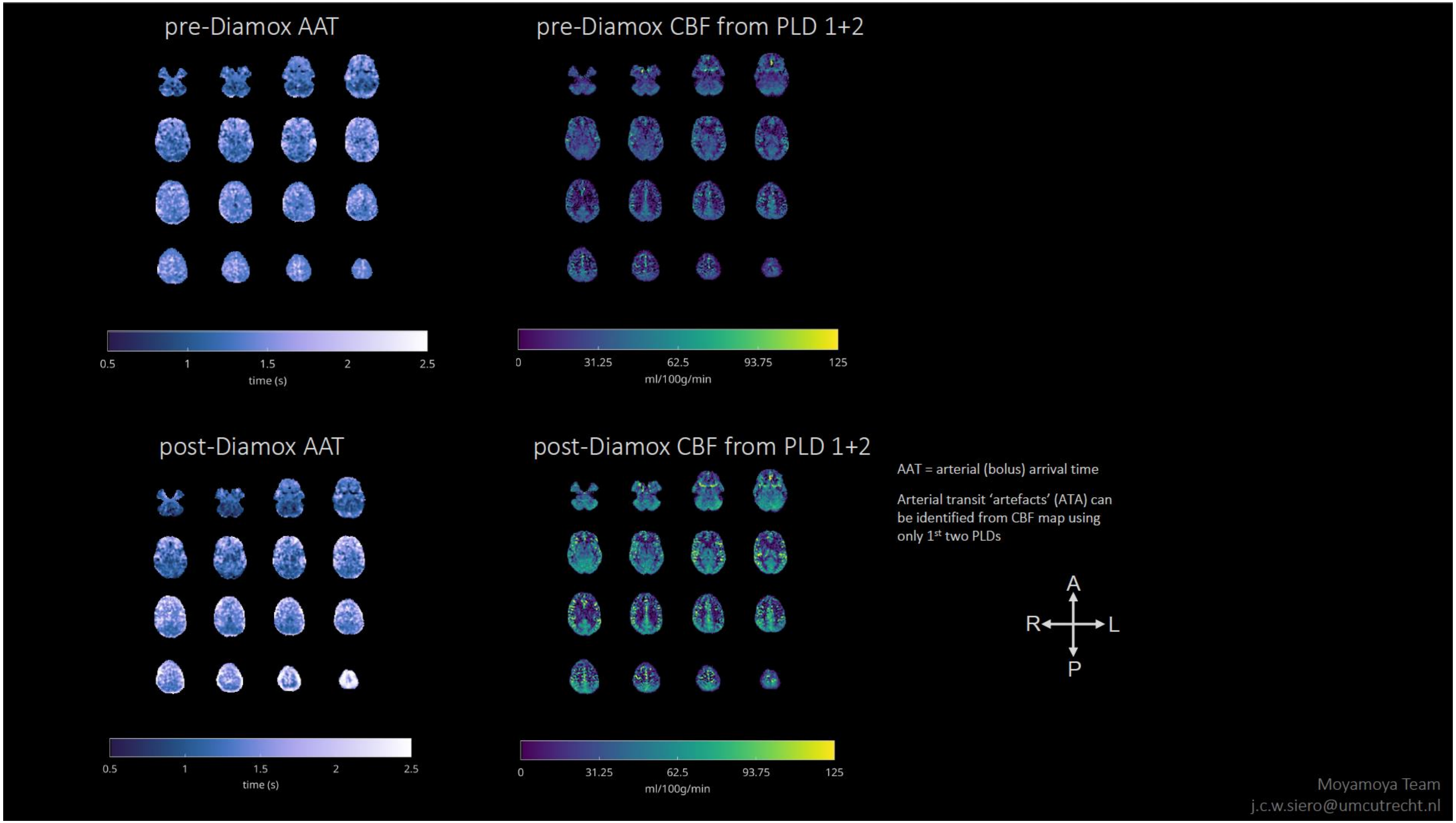

Figure S4 (continued).

Insights Imaging (2025) Uniken Venema SM, Bhogal A, Dankbaar JW, et al.

Table S5: Characteristics of the patient population.

	All (n=104)	Pediatric (n=47)	Adult (n=57)
Age (mean)	30.3	12.9	44.7
Male	35 (33.7 %)	13 (27.7 %)	22 (38.6 %)
Diagnosis			
Moyamoya	82 (78.8 %)	43 (91.5 %)	39 (68.4 %)
Intracranial stenosis due to other cause	22 (21.2 %)	4 (8.5 %)	18 (31.6 %)
Bilateral stenosis on angiography	71 (68.3 %)	34 (72.3 %)	37 (64.9 %)
Anesthesia during scanning	17 (16.3 %)	14 (29.8 %)	3 (5.3 %)
Previous surgery			
Direct MCA-STA bypass	14 (13.5 %)	6 (12.8 %)	8 (14.0 %)
Indirect revascularization	9 (8.7 %)	6 (12.8 %)	3 (5.3 %)
Both direct and indirect revascularization	10 (9.6 %)	8 (17.0 %)	2 (3.5 %)
Other	1 (1.0 %)	0 (0 %)	1 (1.8 %)
None	70 (67.3 %)	27 (57.4 %)	43 (75.4 %)

MCA = middle cerebral artery; STA = superficial temporal artery.
